# “YOU BECOME LESS OF A PERSON”: BEING UNNEEDED AND THE POLITICAL ECONOMY OF LONELINESS IN LATER LIFE

**DOI:** 10.1093/geroni/igad104.0617

**Published:** 2023-12-21

**Authors:** Ad Maulod, Malcolm Ravindran, Angelique Chan

**Affiliations:** Duke-NUS Medical School, Singapore, Singapore; Duke-NUS Medical School, Singapore, Singapore; Duke-NUS Medical School, Singapore, Singapore

## Abstract

Loneliness is a significant public health concern among older persons and refers to the discrepancy between actual and desired forms of social connection. Loneliness is often characterized in terms of (i) lacking companionship, (ii) being left out and (iii) feeling isolated. In comparison, older persons’ experience of being unneeded is relatively underexamined as a construct of loneliness. Being unneeded connects to constrains in attaining social recognition and underpins the complex dynamics of social exchange practices. This study explores the relationship between being unneeded and loneliness in later life, focusing on the impact on one’s identity and ability to form connections with others. We conducted in-depth interviews with 44 older persons in Singapore with varying experiences of loneliness. Using a life-course perspective, participants narrated their experiences of loneliness across domains of family, friendships, work, and community. Loneliness, attributed to being unneeded, features more prominently in later life and pertains to perceived limited personal mastery, negative ageing perceptions, role-loss, life transitions and declines in health. Experiences of being unneeded is embedded in older persons’ beliefs that their self-worth and social recognition is tied to individual strive and diligence– an ideology of meritocracy that has been perpetuated by the Singapore state. Studies have shown how beliefs in meritocracy are associated with lowered likelihood of loneliness among older persons. The Singapore story indicates otherwise – loneliness prevails when older persons with declining resources struggle to find a place in a society which accords worth and recognition to one’s productivity and contribution to the nation.

